# Correspondence analysis, spectral clustering and graph embedding: applications to ecology and economic complexity

**DOI:** 10.1038/s41598-021-87971-9

**Published:** 2021-04-26

**Authors:** Alje van Dam, Mark Dekker, Ignacio Morales-Castilla, Miguel Á. Rodríguez, David Wichmann, Mara Baudena

**Affiliations:** 1 Copernicus Institute of Sustainable Development, Utrecht University, Utrecht, The Netherlands; 2 Centre for Complex Systems Studies, Utrecht University, Utrecht, The Netherlands; 3 Department of Information and Computing Sciences, Utrecht University, Utrecht, The Netherlands; 4 GloCEE-Global Change Ecology and Evolution Group, Department of Life Sciences, University of Alcalá, Alcalá, Spain; 5 Institute for Marine and Atmospheric Research Utrecht, Utrecht University, Utrecht, The Netherlands; 6 National Research Council of Italy, Institute of Atmospheric Sciences and Climate (CNR-ISAC), Turin, Italy

**Keywords:** Biogeography, Ecological networks, Mathematics and computing, Applied mathematics, Scientific data

## Abstract

Identifying structure underlying high-dimensional data is a common challenge across scientific disciplines. We revisit correspondence analysis (CA), a classical method revealing such structures, from a network perspective. We present the poorly-known equivalence of CA to spectral clustering and graph-embedding techniques. We point out a number of complementary interpretations of CA results, other than its traditional interpretation as an ordination technique. These interpretations relate to the structure of the underlying networks. We then discuss an empirical example drawn from ecology, where we apply CA to the global distribution of Carnivora species to show how both the clustering and ordination interpretation can be used to find gradients in clustered data. In the second empirical example, we revisit the economic complexity index as an application of correspondence analysis, and use the different interpretations of the method to shed new light on the empirical results within this literature.

## Introduction

Many systems in natural and social sciences are characterized by high dimensional data sets describing the interactions between the objects of study. Such data can be analyzed by using statistical methods that reduce their complexity by identifying the low-dimensional structures that define the systems’ main features. Identifying these structures enables visualization of the data in two or three dimensions, and can be used in further analysis to gain insight and understanding of the dynamics underlying the system.

A frequently used method to describe the interactions between components in a system starts with collecting data on the joint occurrence of two types of variables in the system. Such data are commonly represented by a contingency table, reporting the frequency with which an outcome of a certain variable is observed in association with the outcome of another variable. A contingency table can be represented as a bipartite network, i.e. a network that connects two sets of nodes (the possible outcomes of each variable), where the edges represent joint occurrences of the outcomes. A typical example of such data sets in ecology are the records of presence (or absence) of species in sampling sites. In economics, a representative data set could be the presence of different types of economic activity (in terms of money or employment) in different regions.

Data represented in that way can be used to infer the associations (or similarities), between nodes of the same type, by considering for example how often species occur together in the same site. In network terms, this entails ‘projecting’ the bipartite network onto one of its node sets, leading to a similarity network^1^.

The bipartite network and the inferred similarity network hold information on the underlying dynamics of the system. For example, ecologists have been investigating the existence of latent variables that determine which species occur in which sites, a practice known as gradient analysis or ordination. These latent variables can be related to environmental variations along a gradient, for example due to latitude or temperature^2–4^. Furthermore, analyzing the similarity networks of species or sites may reveal the existence of multiple subsystems or clusters, such as distinct communities of species or regions with distinct species compositions^5–7^. More recently, economists have also started to study the latent structures underlying countries’ economies by leveraging network analysis to infer measures of economic complexity based on the geographical co-occurrence of products^8–10^.

The analysis of contingency tables is the domain of a statistical method called correspondence analysis (CA). Given a contingency table, CA returns a set of ‘axes’, which, analogously to the components in principal component analysis (PCA), are used to represent the data in a lower-dimensional space, such that the distances between the data points represent the associations between them^11^. These axes can also be used to produce rankings or ordinations of the objects under consideration. Dating back to the seminal statistical work of Hirschfeld in the 1930s^12^, CA was developed in France by Benzécri^13^, and became widespread thanks to a classical paper by Hill, which promoted its use especially for ecological data^14^. CA has been used extensively since the 80’s, being as common in ecological papers as PCA. Since then, CA has been reinvented many times across different fields, leading to a plethora of different names, interpretations and applications of the method^11,15,16^. What we discuss in the present paper is in fact known also as ’simple correspondence analysis’.

The representation of a contingency table as a bipartite network shows that CA can also be used for network analysis. In fact, it can be shown that CA is mathematically equivalent to network methods such as clustering and graph embedding techniques^17,18^ (note that “graph” is another word for network). The equivalence between CA and such network methods is not a simple matter of reinventing the wheel. Since each of the methods is derived with different underlying motivations (ordination, dimensionality reduction or clustering), the results of applying these methods can be interpreted in multiple ways. In this paper, we aim to raise awareness about these different interpretations, stressing that the outcome of CA can be interpreted at the same time as latent variables underlying the data, as node labels indicating cluster membership in a network, and as coordinates that represent the data points in a low-dimensional Euclidean space. By clarifying the relations between these three interpretations, we aim to aid practitioners in the interpretation of both CA and network analysis. We do so by revisiting CA from a network perspective and by providing both stylized and empirical examples to illustrate how these methods can be applied in practice. We will discuss the three alternative derivations of CA.

First, we discuss CA as a form of ‘canonical correlation analysis’^19^, motivated here as a way to find latent variables that drive the connections in a bipartite network^1^. Second, we discuss the interpretation of CA as a spectral clustering algorithm applied to the network of similarities derived from the bipartite network^20^. Third, we discuss CA as a method of graph embedding applied to the similarity network^18^. Each approach leads to a complementary interpretation of the same set of eigenvectors and eigenvalues that result from applying CA.

We illustrate the different interpretations of CA by applying it to a number of stylized networks, showing how the eigenvalues and eigenvectors that result from CA relate to their structure. When the similarity network inferred from a bipartite network consists of a single cluster, the axes resulting from CA can be interpreted as a gradient underlying the data, leading to an ordination of the nodes. However, when the network consists of multiple weakly connected clusters, the CA axes hold information on the clustering structure of the underlying network, showing for each node to which cluster it belongs. Based on these examples, we propose to use CA to cluster the data first before applying it as an ordination method within each cluster, when performing gradient analysis on data containing multiple clusters.

We illustrate these ideas by analysing two empirical examples, drawn from ecology and economics. The proposed methodological approach is available as an R package “SCCA”,  which can be retrieved at Ref.^21^(also available at GitHub: https://github.com/UtrechtUniversity/SCCA). In the first example, we apply CA to an ecological dataset describing the global geographical distribution of Carnivora species, with the objective of finding gradients that reflect drivers of the species distributions. Interpretation of CA as a clustering algorithm motivates dividing the data into subsets, leading to the identification of meaningful bioregions. Applying CA to each bioregion separately results in identification of ecological gradients within those regions.

The second example is drawn from economics, where CA was recently reinvented as a way to analyze bipartite networks under the name of the ‘Economic complexity index’ (ECI). The ECI is used to infer a ranking of countries based on the products they export which is associated to their economic productivity^8,10^. Here we review the ECI from the perspective of CA, partly building on the earlier work of Ref.^10^, which already noticed the equivalence of ECI with CA, spectral clustering and graph embedding. We show how the different interpretations of the mathematics behind CA can help in interpreting economic complexity; besides, we focus in particular on the interpretation of higher order eigenvectors and eigenvalues, which were hitherto not considered in the context of economic complexity.

## Interpreting CA

Let us first describe the setting and introduce notation. The main object of analysis is a matrix *A* (a contingency table with $n_r$ rows and $n_c$ columns) that contains the counts of two variables. A common example from ecology is that $A_{ij}$ contains some measure of abundance of species *i* (rows) in sampling site *j* (columns). The matrix *A* can also be a binary incidence matrix, containing either the presence (1) or absence (0) of species in sites. The matrix *A* can be interpreted as the bi-adjacency matrix of a bipartite network that connects species to sites. The network contains $n_r$ nodes on one side (the species, given by the rows of *A*, indexed by *i*), and $n_c$ nodes on the other side (the sites, given by the columns of *A*, indexed by *j*). In general, we will refer to the two sets of nodes as row nodes and column nodes, respectively. The degree of a row node *i* is defined by the row sum $r_i = \sum _j A_{ij}$, which gives the total abundance of species *i* in all sites. Likewise, the degree of a column node *j* is defined as the column sum $c_j = \sum _i A_{ij}$, which gives the total abundance of species in a site *j*. The degrees of the row and column nodes are given by the vectors $\mathbf {r}= (r_1, r_2, \dots , r_{n_r})^T$ and $\mathbf {c}= (c_1,c_2,\dots ,c_{n_c})^T$. We further define two square matrices, $D_r$ ($n_r \times n_r$) and $D_c$ ($n_c \times n_c$) as the diagonal matrices that have $\mathbf {r}$ and $\mathbf {c}$ on the diagonal, respectively. The sum $n = \sum _{ij} A_{ij}$ gives the total number of occurrences in the table (in the case of a species-site example, the total abundance of species).

### CA as canonical correlation analysis

One of the first derivations of CA was obtained by applying canonical correlation analysis to categorical variables^12,19,22^. Here we follow the derivation in Ref.^1^ (Chapter 9), where CA is derived as an application of canonical correlation analysis applied to a bipartite network, and to which we refer for further details. For ease of explanation, we will assume the network is defined by a binary presence-absence matrix (i.e. the network is unweighted), but the result generalizes to any contingency table (i.e. weighted bipartite networks).

The aim is to assign a ‘score’ to each node in the network, under the assumption that row and column nodes with similar scores connect to each other. Hence, connected nodes get assigned similar scores, and the scores can be thought of as a *latent variable* that drive the formation of links in the network. In ecology, such latent variables are referred to as *gradients*^2,3^. Considering a bipartite network describing the occurrence of species in a set of sites, for example, the resulting scores may reflect some variable determining why species locate in specific sites, such as the temperature preference of a species and the temperature at a site. In practice, the interpretation of a gradient resulting from application of CA can be verified by correlating it with known environmental variables (e.g. data on the temperature of each site).

Mathematically, such gradients can be inferred from the edges of the bipartite network. Recall that for a presence-absence matrix, the total number of edges in the bipartite network is given by $n = \sum _{ij} A_{ij}$. Let us construct a vector $\mathbf {y}_r$ of length *n* that contains, for each edge, the scores of the row node it connects to, and a vector $\mathbf {y}_c$ of length *n* that contains, again for each edge, the score of the column node it connects to. Given the assumption that edges connect row nodes and column nodes with similar scores, the node scores can be found by maximizing the correlation between $\mathbf {y}_r$ and $\mathbf {y}_c$, so that the row- and column scores for each edge are as similar as possible. Denoting the vector of length $n_r$ containing the row scores by $\mathbf {v}$ and the vector of length $n_c$ containing the column scores by $\mathbf {u}$, this leads to the optimization problem

1 $$\begin{aligned} \max _{\mathbf {v}, \mathbf {u}} \mathrm {corr}(\mathbf {y}_r,\mathbf {y}_c). \end{aligned}$$

In order to obtain standardized scores, the constraints that $\mathbf {y}_r$ and $\mathbf {y}_c$ have zero mean and unit variance need to be added. Solving this problem using Lagrangian optimization, the solution is given by

2 $$\begin{aligned} D_r^{-1}A D_c^{-1} A^T \mathbf {v}&= \lambda \mathbf {v}\nonumber \\ D_c^{-1}A^T D_r^{-1} A \mathbf {u}&= \lambda \mathbf {u}. \end{aligned}$$

The score vectors $\mathbf {v}$ and $\mathbf {u}$ can thus be found by solving an eigenvector problem. Following Ref.^1^ , they are subject to the constraint that $|\mathbf {v}|=|\mathbf {u}|=1$. The general interpretation of the elements of $\mathbf {v}$ and $\mathbf {u}$ is as follows. Each row node (of *A*) is represented in $\mathbf {v}$, and each column node is represented in $\mathbf {u}$. The smaller the difference between the values of two row (column) nodes in $\mathbf {v}$ ($\mathbf {u}$), the more similar these nodes are. The similarity among row nodes that is reflected in $\mathbf {v}$ vectors arises because they are connected to a similar set of column nodes in the original bipartite network (vice versa for similarities in $\mathbf {u}$). In literature, this is referred to as row nodes being similar because of their similar ‘profile’^11,23^, and reciprocal averaging defines exactly how the scores are calculated in terms of the profiles and reduces to the same set of equations^15^. Both matrices on the left-hand side of Eq. (2) are row-stochastic and positive definite, and have identical eigenvalues that are real and take values between 0 and 1. Assuming that we have a connected network, sorting the eigenvalues in decreasing order leads to $1=\lambda _1 > \lambda _2 \dots \ge 0$.

It can be shown that the correlation between $\mathbf {y}_r$ and $\mathbf {y}_c$ for a given set of eigenvectors $\mathbf {v}$ and $\mathbf {u}$ is given by their corresponding eigenvalue, so that $\lambda = \mathrm {corr}^2(\mathbf {y}_{r},\mathbf {y}_{c})$. Note that the correlations between the row and column vectors can be negative, meaning that merely the absolute value of the correlation between $y_r$ and $y_c$ is related to the (square root) of the eigenvalues. Iterative approaches to extract potential negative correlations exist in literature^24^. The node scores leading to the highest correlation are thus given by the eigenvectors associated with the largest eigenvalue. However, the eigenvectors corresponding to $\lambda _1$ have all constant values and represent the trivial solution in which all row nodes and all column nodes have equal scores (leading to a perfect correlation). This trivial solution does not satisfy the condition that the scores have to be centered, and thus it must be rejected. The solution to Eq. (1) is thus given by the eigenvectors $\mathbf {v}_2$ and $\mathbf {u}_2$, corresponding to the second largest eigenvalue $\lambda _2$, which corresponds to the square root of the (maximized) correlation. We notice here that this derivation leads to analogous results than observed in classical derivations of CA, where the matrix A is centered both with respect to the rows and to the columns.

The second eigenvectors $\mathbf {v}_2$ and $\mathbf {u}_2$ hold the unique scores such that row- and column nodes with similar scores connect to each other. The second eigenvalue $\lambda _2$ indicates to what extent the row- and column scores can be ‘matched’, where high values (close to 1) indicate a high association between the inferred scores (the gradient) and the structure of the network.

The higher order eigenvectors in Eq. (2) and their eigenvalues are solutions to Eq. (1) with the additional constraint that $\mathbf {y}_r$ and $\mathbf {y}_c$ are orthogonal to the other solutions. The vectors $\mathbf {v}_3$ and $\mathbf {u}_3$, for example, may represent other variables that drive the formation of links (e.g. precipitation, primary productivity, etc.) on top of the gradients described by $\mathbf {v}_2$ and $\mathbf {u}_2$. We note that, differently from notation in some CA literature, we here denote the *k*-th non-trivial eigenvector with the subscript *k*+1.

### CA as a clustering algorithm

A completely different approach shows that the eigenvectors $\mathbf {v}_2$ and $\mathbf {u}_2$ (i.e. the second eigenvectors in Eq. (2)) can also be interpreted as cluster labels, obtained when identifying clusters in the network of similarities that is derived from the bipartite network.

A similarity network can be constructed from a bipartite network by ‘projecting’ the bipartite network onto one of its layers (either the row nodes or the column nodes) through stochastic complementation^18^. Projecting the bipartite network defined by *A* onto its row layer leads to the $n_r \times n_r$ similarity matrix $S_r = A D_c^{-1} A^T$. The entries of $S_r$ represent pairwise similarities between row nodes of *A*, based on how many links they share with the same column node, weighted for the degree of each column node. Similarly, the $n_c \times n_c$ similarity matrix $S_c = A^T D_r^{-1} A$ defines the pairwise similarities between the column nodes of *A*.

Identifying clusters in the similarity network can be done by minimizing the so-called ‘normalized cut’^20^. The normalized cut assigns, for a given partition of a network into *K* clusters, a score that represents the strength of the connections between the clusters for that partition. A partition can be described by assigning a discrete cluster label to each node. Hence, minimizing the normalized cut is equivalent to assigning a cluster label to each node in the network in such a way that the clusters are minimally connected. Finding the discrete cluster labels that minimize the normalized cut in large networks is in general not possible^20^. However, a solution of a related problem can be obtained when the cluster labels are allowed to take continuous values as opposed to discrete values. Solutions of this ‘relaxed’ problem can be interpreted as continuous approximations of the discrete cluster labels.

Minimizing the normalized cut in $S_r$ leads to the generalized eigensystem^20^

3 $$\begin{aligned} (D_r - S_r) \mathbf {v}= \tilde{\lambda } D_r \mathbf {v}, \end{aligned}$$

where the entries of the generalized eigenvector $\mathbf {v}_2$ corresponding to the second smallest eigenvalue $\tilde{\lambda }_2$ of Eq. (3) hold the approximate cluster labels of the optimal partition into two clusters. It is easily shown that generalized eigenvectors in Eq. (3) are exactly the eigenvectors of Eq. (2), where the eigenvalues are related by $\tilde{\lambda }_k = 1 - \lambda _k$, where $k=1,2,\dots ,n_r$ (see “Suppl. Material A”).

The matrix $L_r = D_r-S_r$ is known as the *Laplacian* matrix of the similarity network defined by $S_r$, and is well known in spectral graph theory^25^. The number of eigenvalues of $L_r$ for which $\tilde{\lambda } = 0$ (or equivalently $\lambda = 1$ in Eq. (2)) denotes the number of disconnected clusters in the network. Each of these ’trivial’ eigenvalues has a corresponding generalized eigenvector that has constant values for the nodes in a particular cluster, indicating cluster membership.

The situation changes when the clusters are weakly connected. The optimal solution for partitioning the similarity network into two clusters is given by the eigenvector $\mathbf {v}_2$ associated to eigenvalue $\lambda _2$. Although continuous, the entries of $\mathbf {v}_2$ can be interpreted as approximations to cluster labels, which indicate for each row node to which cluster it belongs. In other words, nodes with high values in this eigenvector (i.e., high scores) belong to one cluster, and nodes with low scores to the other. A discrete partition can then be obtained from the approximate (continuous) cluster labels by discretizing them, for example by assigning all negative values to one cluster and all positive values to the other^26^. The corresponding eigenvalue $\lambda _2$ represents the quality of the partitioning, as determined by the normalized cut criterion. High values are indicative of a network that can be well partitioned into two clusters (two totally disconnected clusters would yield eigenvalues $\lambda _1 = \lambda _2=1$), whereas lower values correspond to a network that is less easily grouped into two clusters (i.e. the resulting clusters are more interconnected).

Finding a partitioning into *multiple*, say *K*, clusters is more involved, where $K\le n_r$ (or $K\le n_c$ if working with column variables). Minimizing the normalized cut for *K* clusters yields a trace minimization problem of which the relaxed solution is given by the first *K* eigenvectors in Eq. (2)^27^. Because the first eigenvector in Eq. (2) is trivial, in practice we only require $K-1$ eigenvectors (i.e., the 2nd, 3rd, ... up to the *K*th). The discrete cluster labels can then be obtained, for example, by running a k-Means algorithm on the matrix consisting of those $K-1$ eigenvectors, a technique that is also known as *spectral clustering*^28,29^. How well the network can be partitioned into *K* clusters is given by the average value of the first *K* eigenvalues, i.e. $\frac{1}{K} \sum _{k=1}^K \lambda _k$^27^.

The clustering approach thus brings an alternative interpretation to CA results. A key observation is that the eigenvalues and eigenvectors in Eq. (2) are directly related to the generalized eigenvectors of the Laplacian of the similarity matrix $S_r$, and thus hold information on the structure of the similarity network. The entries of the second eigenvector $\mathbf {v}_2$ can be interpreted as the approximate cluster labels of a two-way partitioning of the similarity network defined by $S_r$. Although at first sight the interpretation of CA scores as cluster labels may seem different from the interpretation as a latent variable described above in “CA as canonical correlation analysis”, note that cluster labels can be seen as latent variables, albeit discrete rather than continuous.

### CA as a graph embedding technique

A third interpretation of the eigenvectors and eigenvalues in Eq. 2 arises from a so-called *graph embedding* of the similarity matrix $S_r$ (or $S_c$). Graph embeddings provide a way to obtain a low-dimensional representation of a high-dimensional network, that are used for example for graph drawing. A graph embedding represents the nodes of a graph as *node vectors* in a space, such that nodes that are ‘close’ in the network are also close in terms of their distance in the embedding. A key feature of these embeddings is that their dimensionality can be reduced in order to obtain a low-dimensional representation of the data, while retaining its most important structural properties (see Ref.^1^, chapter 10 for an overview of graph embedding techniques).

As noted by several authors, CA is equivalent to graph embedding in the case of a similarity matrix obtained through stochastic complementation. For example, computing a 1-step diffusion map of the similarity matrix $S_r$ leads exactly to the eigenvectors of Eq. (2)^18,30^. Also, the graph embedding using the Laplacian eigenmap has been shown to be equivalent to graph partitioning using the normalized cut, which is in turn equivalent to CA^31^. CA-specific type of embedding is based on the chi-square statistics and it is thus Euclidean.

Embedding the similarity network $S_r$ in a $(K-1)$-dimensional space yields an ‘embedding matrix’ $X_r \in \mathbb {R}^{n_r \times K-1}$ (known in CA-related literature as ’principal coordinates’). Each row of $X_r$ represents a node of $S_r$ as a ‘node vector’ in the embedding. The rows of $X_r$ can be seen as components of $(K-1)$-dimensional basis vectors that span the embedding, and are identical to what is referred to as the ‘axes’ in CA. Every entry $X_{i,k}$ represents the coordinate of row node *i* on the *k*’th basis vector, and can be seen as the ‘score’ of *i* on the *k*’th CA axis. An embedding matrix of $S_r$ can defined as $X_r = [\sqrt{\lambda _2} \mathbf {v}_2, \dots , \sqrt{\lambda _K} \mathbf {v}_{K}]$, where the vectors $\mathbf {v}_k$ are the eigenvectors defined in (2), and each of them is weighted by the square root of their corresponding eigenvalue. We will refer to columns of the embedding matrix as ‘CA-axes’, given by $\mathbf {x}_k = \sqrt{\lambda _k}\mathbf {v}_k$ (with $\mathbf {x}_2$ being the ’first CA axis’, and so on).

The axes are constructed in such a way that they capture the largest amount of ‘variation’ or ‘inertia’ in the data, which is given by their corresponding eigenvalue^11^. The sum of all the eigenvalues gives the total variation in the data (in CA, this is referred to as the *total inertia*). CA decomposes the total variation in such a way that the first axis captures a maximal part of the variation, the second a maximal part of the remaining variation, and so on. A low-dimensional embedding that preserves the maximal amount of variation can thus be obtained by discarding the eigenvectors corresponding to smaller eigenvalues. The ‘quality’ of the embedding can then be expressed as the share of the total variation that is preserved in the embedding.

A typical way of presenting CA results is by showing the first two coordinates of each row (or column) node, i.e. plotting $\mathbf {x}_2$ against $\mathbf {x}_3$, which is usually referred to as a ’correspondence plot’. Since the first two axes capture a maximal amount of inertia, such a plot is in a way the optimal two-dimensional representation of the data that captures the relations between the rows (or columns) of *A*. The distances between points in the correspondence plot approximate the similarities between nodes. How well the correspondence plot represents the similarities is given by the percentage of variation explained by the first two axes.

Each axis can be interpreted as a latent variable that account for part of the total variation in the data. Since the axes in the embedding are given by a scaled version of the eigenvectors discussed above in “CA as canonical correlation analysis”, the interpretation of the eigenvalues as the amount of variation explained is complementary to the interpretation as the correlation between row and column scores which we also introduced above in “CA as canonical correlation analysis”. Furthermore, the axes spanning the *K*-dimensional embedding are exactly the generalized eigenvectors that follow from minimizing the normalized cut for *K* clusters^31^. Indeed, when there are clear clusters in the similarity network, they will show up in the embedding space as separate groups of points.

Summarizing, we find three interpretations of CA axes and their corresponding eigenvalues: as latent variables that drive the formation of links in the bipartite network, as approximate clusters labels of a bi-partition of the similarity network, and as coordinates of an embedding of the similarity network. The different derivations of CA and their interpretations are summarized in Table 1.

**Table 1:** Different interpretations of the eigenvectors and eigenvalues resulting from CA.

Method	Interpretation eigenvectors	Interpretation eigenvalues
Gradient analysis using canonical correlation analysis	Latent variable	Strength of correlation between row and column scores
Graph partitioning using the normalized cut	Approximate cluster labels	Quality of the partitioning (given by normalized cut)
Dimensionality reduction using graph embedding	Coordinates in the embedding space	Variation explained

## Stylized examples

**Figure 1:**
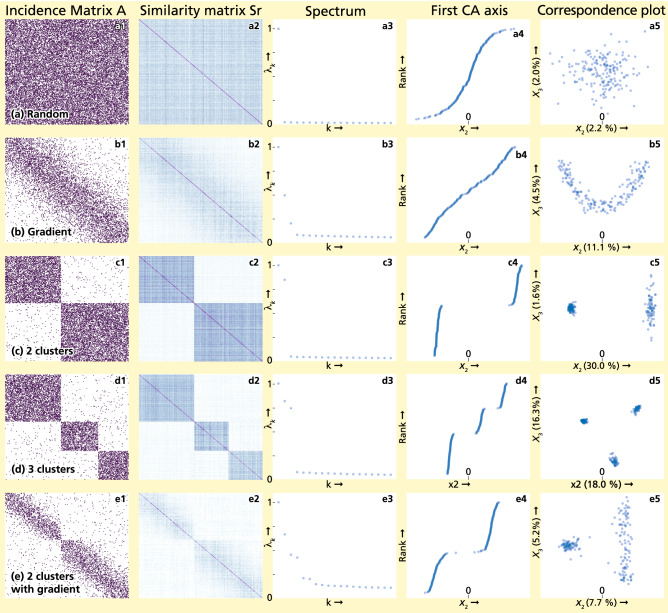
Stylized examples of five network structures and their corresponding spectra and eigenvalues. From top to bottom: a random bipartite network **(a)**, a network with a clear gradient **(b)**, a network consisting of two weakly connected clusters **(c)**, a network consisting of three weakly connected clusters **(d)**, and a network consisting of two weakly connected clusters containing a gradient **(e)**. From left to right: the $n_c\times n_r$ incidence matrix *A*, the $n_r\times n_r$ similarity matrix $S_r$ describing pairwise similarities between the rows, the spectrum of the first 20 eigenvalues, the first CA axis $\mathbf {x}_2$ (dimension $n_r$), and the correspondence plot of the first and second CA axes $\mathbf {x}_2$ and $\mathbf {x}_3$, respectively, with the percent variation explained for both axes.

In the following, we illustrate the interpretations found above by applying CA to a set of stylized networks: a random bipartite network (Fig. 1 panels a), a network defined by an incidence matrix with a band-diagonal structure (Fig. 1 panels b), networks with two or three weakly connected clusters (Fig. 1 panels c,d), and a network with two clusters that each have a band-diagonal structure (Fig. 1 panels e).

The columns of Fig. 1 show from left to right: the incidence matrix *A* (where the $n_r$ rows and the $n_c$ columns are sorted according the their scores in $\mathbf {v}_2$ and $\mathbf {u}_2$), the similarity matrices of the row nodes $S_r$ ($n_r \times n_r$), the spectrum of eigenvalues of the row-normalized similarity matrices, the (scaled) eigenvector $\mathbf {x}_2$ corresponding to the second largest eigenvalue (the first CA axis) and the correspondence plot, which shows the two-dimensional embedding as given by the values of the first two CA axes (i.e., the second and third eigenvectors, $\mathbf {x}_2$ and $\mathbf {x}_3$).

For the random bipartite network (Fig. 1a1), the single trivial eigenvalue $\lambda _1=1$ indicates that the similarity network *S* consists of a single connected component (Fig. 1a3). Its corresponding eigenvector has constant values (not shown). The second eigenvector shows the node scores that maximize the correlation between row and column nodes (Fig. 1a4). Since the network is random, this correlation is low ($\sqrt{\lambda _2} = \sqrt{0.02} = 0.14$), indicating the absence of a clear underlying structure that can be captured in a single variable. Accordingly, the correspondence plot (Fig. 1a5) does not show any particular structure, and each axis explains a limited amount (approximately $2\%$) of the total variation in the data .

Different patterns are observed in a network with a clear band-diagonal pattern (Fig. 1b1). This pattern is indicative of a gradient underlying the structure of the bipartite network, since high-score row nodes (on the right-hand side of the matrix) connect to high-score column nodes (on the bottom of the matrix) and vice versa. Indeed, the spectrum contains, next to the trivial eigenvalue ($\lambda _1=1$) two eigenvalues that are larger than the others (Fig. 1b3). The strength of the correlation between row nodes and column nodes is given by $\sqrt{\lambda _2} = \sqrt{0.5}=0.71$, and the gradient for the row nodes is given by the axis $\mathbf {x}_2$ shown in Fig. 1b4). The third eigenvalue $\lambda _3$ is much smaller than the second, but slightly larger than the subsequent eigenvalues. The correspondence plot (Fig. 1b5) shows that the corresponding axis $\mathbf {x}_3$ is approximately a quadratic function of the first. This is a statistical artefact known as the ‘arch effect’ (these type of axes were referred to as ‘polynomial axes’ by^15^). Such solutions arise because a quadratic function of the ‘true’ gradient also leads to positive correlation, and is orthogonal to the solution given by $\mathbf {x}_2$^32^. The solution thus contains little extra information on top of what is already reported by the second eigenvector and can thus be ignored in practice^4^.

In the subsequent example, the network is constituted by two weakly connected random bipartite networks, so that the similarity network presents two clusters (Fig. 1c1). This represents a slightly perturbed case of the situation in which the clusters are totally disconnected. Hence, the second eigenvalue (Fig. 1c3) is close to one, and, unlike the situation in Fig. 1b4, its corresponding eigenvector (Fig. 1c4) shows a clear separation between two well-separated sets of values. As we explained in “CA as a clustering algorithm”, these entries may be interpreted as approximations to cluster labels, identifying the two clusters. The higher order eigenvectors identify axes that show variation within each of the two clusters. For example, the correspondence plot (Fig. 1c5) shows that the second axis $\mathbf {x}_3$ only varies in one of the clusters (as identified by $\mathbf {x}_2$) and is approximately constant for nodes in the other cluster.

A similar situation is found for a network constituted of three weakly connected random bipartite networks (Fig. 1d1). Since there are now three clusters, both eigenvectors $\mathbf {v}_2$ and $\mathbf {v}_3$ are associated to eigenvalues that are close to 1 (Fig. 1d3). While $\mathbf {x}_2$ only identifies a two-way partition, both axes together clearly identify the three clusters, as shown in the correspondence plot (Fig. 1d5).

Finally, we consider what happens when a network consists of two weakly connected clusters that each have a clear gradient, shown by their band-diagonal pattern in the incidence matrix (Fig. 1e1). The spectrum shows four eigenvalues that are far away from zero (Fig. 1e3). The first two eigenvalues identify two well-defined clusters, and the second eigenvector clearly separates the two clusters (Fig. 1e4). The third and fourth eigenvalues correspond to the gradients within each of the clusters. The correspondence plot shows variation in one of the clusters (Fig. 1e5). The gradient of the other cluster is contained in the fourth axis (not shown).

## Clustering versus ordination

The different interpretations of CA axes and their eigenvalues can help interpret results when applying CA to real data. In particular, the entries of the CA axes can be organized in different ways: either they vary smoothly, representing a continuous gradient, or their values are strongly separated and discontinuous, representing a clear partitioning of the data. Hill termed these two types of axes as ‘seriation’ axes and ‘nodal’ axes, respectively^15^. Early applications of these two meanings can be found in Greenacre’s suggestion to use the discontinuous CA axes to help interpreting results of clustering analyses performed with other methods^33^, and in Hill’s TWINSPAN, a popular tool of analysis in plant ecology, used to classify both plant species and their occurrence sites using the first two axes of CA only^34^.

Axes with smoothly varying values are typically used to construct an ordination of the nodes under consideration (in some graph partitioning applications, on the other hand, these axes are discarded when finding a discrete partitioning of the data^20^). Axes containing groups of approximately constant values, indicating cluster membership, can be used to identify discrete clusters, for example by using k-Means. The eigenvalue corresponding to an axis is indicative of both the strength of the gradient and how well the eigenvector partitions the similarity network.

While in the stylized networks presented above the number of clusters and/or the presence of a gradient was imposed and thus known beforehand, this is typically not the case in practice. Real data may consist of weakly connected clusters that may or may not have underlying gradients. This can make results hard to interpret, especially when using noisy data. If the objective of applying CA is to find gradients underlying the data, the CA axes with their continuously varying values are the subject of interest. In datasets consisting of multiple clusters, such gradients will be present only within each cluster, and thus found in the higher order axes (as in Fig. 1e). However, the higher order axes will be increasingly affected by noise^20^. Therefore, we propose to separate the clusters in the data prior to finding gradients, so that each cluster can be analyzed separately, leading to better identification of the within-cluster gradients.

Since the distances are Euclidean, the clusters can be identified by applying k-Means clustering to the embedding of the similarity network^28,29^. This requires estimating the number of clusters beforehand. A correct estimation of this number is important, since imposing too many clusters (i.e. including axes representing gradients) may lead to artificial cuts in the data. A common approach for determining the number of clusters *K* is the ‘eigengap heuristic’^29^, which is based on the fact that the number of connected components is given by the number of eigenvalues equal to one. The issue of how many axes to use is discussed elaborately in literature (see e.g. Ref.^23^, where other approaches are mentioned as well). For the case of a network with weakly connected clusters, the number of clusters can be estimated by counting the eigenvalues that are close to one. More specifically, we count the number of eigenvalues in the spectrum that precede the largest difference between two subsequent eigenvalues, i.e. the so-called ‘eigengap’. For example, the spectra of the example networks with two or three clusters (Fig. 1c3,d3) clearly shows how the positions of the eigengaps mark the number of clusters present.

We hereby define a possible procedure to find gradients in data. When applying CA to a given dataset, we determine whether the data contain multiple clusters and, if so, how many. This can be done in different ways, for example, by using the eigengap heuristic. We then separate the clusters, for example by applying *K*-means. The procedure should then be repeated for each individual cluster, since each of them may also consist of multiple clusters. The repetition should stop when no cluster can further subdivide. Each cluster now represents a well-connected similarity network, of which the CA axes represent continuous gradients. The procedure described in this paragraph is built into our SCCA R package^21^. We note that there are several alternative packages to run CA in R, e.g. ‘ca’^35^, ‘CAvariants’^36^, ‘anacor’^37^, ‘FactoMineR’^38^ or ‘vegan’^39^, see Ref.^36^ for a list. The added value of the SCCA package is the implementation of the eigengap heuristic followed by k-means to cluster the data prior to applying CA.

In the following, we explore the effects of the preceding considerations when applying CA to two different empirical datasets. The first example consists of an ecological dataset that contains clear clusters, and we aim to identify gradients within those clusters, applying the procedure described above. We use the eigengap heuristic to obtain a clustering of the data and subsequently analyze the clusters using CA. In the second example, we apply CA to data on international trade, and illustrate how the alternative interpretations of CA axes and their eigenvalues can help elucidate the economic complexity index.

## Applying CA

### Example I: carnivora biogeography

**Figure 2:**
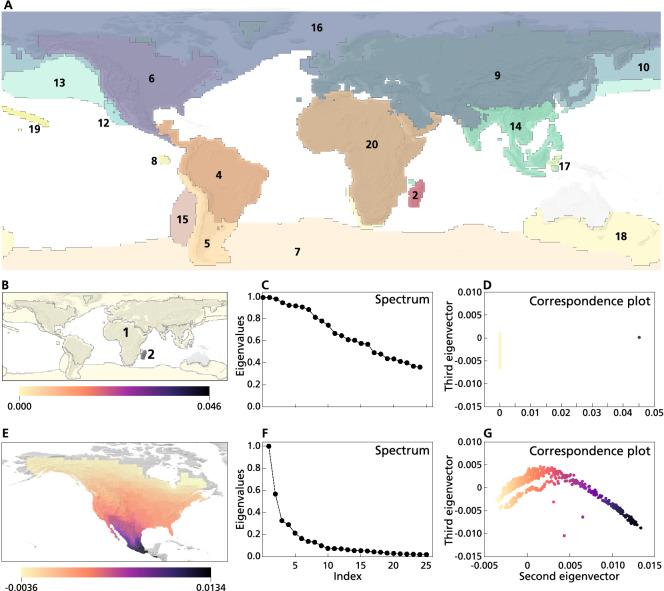
Applying CA to ecological data: the global geographical distribution of the Carnivora mammalian order. Panel **(A)**: Map showing the spatial distribution of the clusters (i.e., bioregions) resulting from application of our heuristic approach to CA. The numbers indicate the resulting clusters. The first split separates Madagascar (2) from the rest of the world (1) (see also panel **B**). The rest of the world cluster is subsequently split into non-nested clusters, of which one is further split into clusters 12 and 13. Panel **(B)**: Spatial distribution of the coefficients in the first CA axis when all data is considered. Madagascar is colored in red (2) and the rest of the world in blue (1). Panel **(C)**: Sorted spectrum for all data, showing two eigenvectors with value equal 1, indicating that there are two fully disconnected clusters. Panel **(D)**: Correspondence plot of first and second CA axes (i.e., 2nd and 3rd eigenvector). Colors indicate the first axis, corresponding to colors in panel **(B)**. The first axis separates the two clusters, but does not show variation within clusters. Panel **(E)**: Spatial distribution of the coefficients in the second eigenvector for the subcluster 6 corresponding to the Nearctic bioregion, in North America. The eigengap is found between the first and second eigenvalues, suggesting that the network does not contain any subclusters **(F)**. Panel **(G)**: The correspondence plot for the first and second CA axes, colored according to panel **(E)**, showing how the first axis separates sites along a clear latitudinal gradient. This analysis was performed using our SCCA R package. Maps were produced with Qgis https://qgis.org/.

To illustrate the application of CA as a method of both clustering and ordination, we applied it to a commonly used dataset in macroecology, i.e., the branch of ecology studying ecological patterns and processes at broad geographical scales, also known (*sensu lato*) as biogeography. The data describe the global geographical distributions of the species of the mammalian order Carnivora^40^. The data are described with a presence–absence incidence matrix, with 288 extant terrestrial and marine species (rows) and 41,580 non-empty sites (columns). The sites represent grid-cells rasterized at a resolution of 0.78 latitudinal degrees. The distributional data were extracted from the mammal range map database Phylacine v1.2^41^, which we downloaded (last accessed in November 2019; https://datadryad.org/stash/dataset/doi:10.5061/dryad.bp26v20) and pruned to only include extant carnivorans. Data were processed in R (R Core Development Team 2014), mostly with our own SSCA package^21^, and mapped in QGIS v2.18.16 (QGIS Development Team 2015).

In this example, we primarily focused on analyzing the sites based on their species composition. Yet, considering that CA simultaneously uncovers patterns for both the rows and the columns of the incidence matrix, we could have as well aimed at analyzing species based on their geographic distributions by applying the same procedure to columns instead of rows (see e.g. Ref.^42^). Application of CA reveals that the dataset consists of multiple clusters. As introduced in “Clustering versus ordination”, we first split the dataset into separate subsets that each consist of a single cluster. These clusters represent sites with similar species composition (termed bioregions). Once we defined the bioregions, we asked whether the sites within them showed any distributional gradient, which would be reflected by the arrangement of sites along “meaningful” CA axes, i.e. axes explaining a considerable amount of variation, as given by the corresponding eigenvalue. If so, it would then be possible to ask whether this gradient would relate to some environmental factor or other spatially patterned process (e.g. distance to main areas of species interchange with other regions^43^). These kinds of questions are common in CA-based ecological investigations and are oftentimes addressed through correlational analyses between meaningful CA-axes and explanatory factors. Since this is well-known practice among ecologists, we did not include this part in the present analysis.

Applying CA to the complete dataset yields two eigenvalues equal to 1, showing that the network consists of two completely disconnected components. The eigenvectors corresponding to these two trivial eigenvalues indicate the membership of each node to one of the two disconnected components, which correspond to Madagascar and to the rest of the world, respectively (see Fig. 2B–D). Malagasy carnivorans belong to the family Eupleplidae and comprise ten species all endemic to Madagascar (i.e. they occur nowhere else), which accounts for this primary differentiation.

We proceed by analyzing the spectrum of each of the identified components separately. Looking at the spectrum of Madagascar, we find the eigengap in the first position, indicating a network consisting of a single cluster. The spectrum of the rest of the world, on the other hand, shows the largest decrease in the eigenvalues (the eigengap) between the 16th and 17th eigenvalue, indicating the existence of at least 16 other bioregions with faunas overlapping to some extent. To obtain these clusters, we follow the spectral clustering approach and apply a k-Means clustering on the embedding matrix defined by the 16 axes corresponding to these eigenvalues.

Applying CA again to each of the resulting 16 clusters, we found that 15 of them were recognized as integral bioregions (regions 4 to 20 in Fig. 2A, with the exception of regions 12-13) and required no further partitioning based on the eigengap heuristic (i.e. the eigengap was found between the first and the second eigenvalues). One of the clusters was split once more based on its spectrum, yielding regions 12 and 13, which comprise the North American Pacific (Fig. 2A).

Although this analysis is only based on the mammalian order Carnivora, and even though it includes both terrestrial and marine species, the bioregions obtained correspond remarkably well to the bioregions first defined by Wallace and supported by recent work on bioregionalization^5^. Besides finding the above described clustering in bioregions, we find informative results on potential gradients of site distribution within the obtained bioregions. In North America, for example, the first CA axis shows a marked north to south gradient (see Fig. 2E–G) that would likely reflect a spatial structuring of species distributions as a response to latitudinal variation in climate.

The first CA axis has been recurrently used in ecology^44^ to conduct either cluster or gradient analyses, which has allowed ecologists and biogeographers to identify regions with similar species composition or to understand how species distributions structure along environmental gradients. However, gradient analyses may only yield interpretable results when the network considered does not consist of multiple clusters. A ‘naive’ application of CA as an ordination method in the example above would have yielded uninterpretable results, as the first axis would have been a one-dimensional representation of a high dimensional data set. Recognizing the clustered nature of the data by considering the whole spectrum and multiple axes allows for splitting the data into homogeneous subsets using the clustering approach, before applying CA as a method for gradient analysis.

### Example II: economic complexity

In the second example, we apply CA to data on international trade, obtained from Harvard’s Growth Lab^45^. We also use data on gross domestic product per capita (GDPpc) in 2016, as given in PPP constant 2017 international dollars, and taken from the World Bank databank https://databank.worldbank.org. From the trade data, we construct a ‘presence-absence’ matrix with 234 countries (rows) and 1239 products (columns), in which a ‘presence’ indicates that a country was a significant exporter of a product in the year 2016 (see Suppl. Material B for an exact description of this procedure). This matrix has been analyzed extensively in relation to economic development^8,9,46–48^. In the literature on economic complexity, the first CA axes of countries and products (the row and column nodes) are known as the economic complexity index (ECI) and product complexity index (PCI), respectively^10^. The ECI has been used as a method of ranking countries by the complexity of their economy, and has become known for its ability to predict the cross-country differences and future growth of countries’ GDPpc^8,9^. The ECI has since been applied to a variety of datasets in economics^49–51^ (see also Ref.^48^ for a review), and beyond^52^.

**Figure 3:**
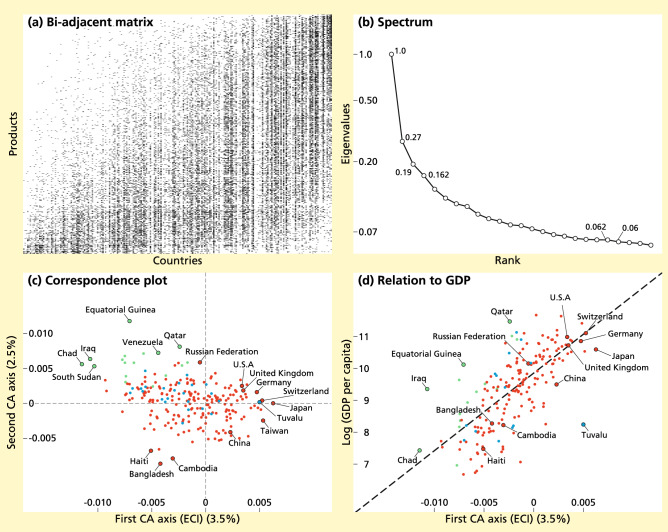
Results of applying CA to the country-product matrix. **(a)**: The country-product incidence matrix shows a triangular structure. Column and rows are sorted by the first CA axes, known as ECI and PCI for the country and the product similarity matrices, respectively. **(b)**: Sorted spectrum for the country-product matrix (log-scale). The slow decay and lack of clear gaps in the spectrum suggests a high-dimensional, homogeneous dataset. Numerical labels are reported for the first four eigenvalues, and for the twenty-first and twenty-second (the small gap between the latter two motivated the choice of a twenty-dimensional embedding in the final part of the analysis) **(c)**: Correspondence plot showing the first (horizontal) and second (vertical) CA axes for the country similarity matrix. The first CA axis is known as the ECI, and explains $3.5\%$ for the total variation. The second axis explains $2.5\%$ of total variation and seems to distinguish countries specializing in garments and textiles from other countries. Colors indicate the obtained clusters when running k-Means with $K=3$ on the embedding spanned by the first 20 CA axes. **(d)**: GDP per capita as a function of the first CA axis (ECI). The dashed line is the linear regression of *log*(*GDPpc*) with ECI ($R^2$=0.49). Colors indicate the same clusters as in **c**). The analysis was performed using our SCCA R package^21^.

Applying CA to the country-product matrix results in an embedding of the country-country similarity network, where similarities are based on the countries’ export portfolios (alternatively, we could have analyzed how products relate to each other in terms of the countries that export them, leading to a variation of the ‘product space’ introduced in Ref.^46^). Figure 3a shows the country-product matrix, where countries and products are sorted by the first CA axis (the ECI and PCI, respectively). The correlation associated with the country and product scores is given by $\sqrt{\lambda _2} = 0.52$. This correlation is an indication to what extent countries with high ECI export products with high PCI and vice versa. The moderate correlation and the triangular shape of the matrix however show that this statement is only partially true, as the lower right side of the matrix shows that typically countries with high ECI also export products with low PCI. A high correlation would imply a more pronounced band-diagonal structure of the country-product matrix.

Moving beyond the first CA axes, we examine the spectrum of eigenvalues, of which the first 25 are shown in Fig. 3b. The spectrum shows no significant gaps in its decay, suggesting that the country-country similarity network does not consist of multiple weakly connected clusters according to the eigengap heuristic. Figure 3b shows the correspondence plot of the first and second CA axes for the country-country similarity network. Expressed as a percentage of total variation, the first axis (the ECI) accounts for $3.5\%$ of the total variation, and the second axis for $2.5\%$, so the correspondence plot captures $6\%$ of the total variation in the country-product matrix. Despite these low figures, indicating weak patterns, we proceeded analyzing these axes to illustrate both the interpretation of ECI as the first axis of CA and to show the extra possibilities that this analysis offers to find structures in data. The distances between countries in the correspondence plot reflect similarities between countries in terms of their export baskets. As expected from the lack of a clear gap in the spectrum, the correspondence plot shows no clearly delineated clusters, suggesting an interpretation of each axis as a continuous gradient. The first axis differentiates between what can be qualitatively identified as low-income countries that mostly export crude oil, such as Chad, Iraq and South-Sudan, on the left-hand side of the plot, and wealthy countries involved in high-tech manufacturing, such as Japan, Taiwan and Switzerland, on the right-hand side. The second axis assigns the highest scores to countries like Equatorial Guinea, Qatar and Venezuela, which can be generally labelled as major oil producers, and assigns low score to countries such as Bangladesh, Cambodia and Haiti, which can be identified by their specialization in textiles and garments.

The separation of low-income and high-income countries by the first CA axis becomes clear when used as a predictor of GDPpc (Fig. 3c), with a linear relationship explaining about 47% of the variance. This relation is interpreted in Ref.^8^ as more complex countries being able to achieve higher levels of GDPpc. Furthermore, countries that are located below the regression line are expected to have high growth rates, as they are less rich than expected given their economic complexity, while countries above the regression line are richer than expected by their economic complexity. The typical interpretation of CA, however, leads to a more agnostic take on the meaning of ECI and its relationship with GDPpc. The ECI reflects a gradient that captures the maximal amount of variation in the data (around $3.5\%$), which in turn can be interpreted as a one-dimensional embedding of the country-country similarity network. In particular, ECI is a measure of similarity rather than ‘complexity’^10^. According to such interpretation, the correlation between ECI and GDPpc shows that countries with similar export baskets also have similar wealth.

Finally, we explore what can be learned from the higher order CA axes of the country-product matrix. Even though the eigengap heuristic does not suggest clearly delineated clusters, the higher order axes may still distinguish (groups of) countries that differ from the other countries in a particular way. To explore this possibility, for illustrative purposes, we present another way to explore the data, beyond the eigengap heuristic. We attempt to identify clusters in a high-dimensional embedding, by running a k-Means algorithm on the 20-dimensional embedding of the country similarity matrix. The number of dimensions is motivated by the slight gap between the twenty-first and twenty-second eigenvalues (Fig 3b). Choosing $K=3$ (again motivated by a small gap in the spectrum) leads to the identification of the three clusters shown in color in Fig. 3c,d (see “Suppl. Appendix C” for an overview of the clusters). The clusters clearly separate: (i) countries whose exports consist almost entirely out of oil, (ii) a number of small island economies, and (iii) the rest of the world. The positions of the countries in each cluster in Fig. 3d suggest that the obtained clustering is able to explain some of the deviations from the relation between GDPpc and the ECI, attributing it to the presence of natural resources or to their unique geographical locations. In this sense, identifying clusters defined by higher order axes may provide a way to remove outliers and reduce noise in the data before identifying a gradient. Recomputing the CA axes within the rest-of-the world cluster yields a noticeable increase of the $R^2$ for the linear relation between GDPpc and the first CA axis ($R^2=0.65$). The two choices for the number of dimensions and clusters, although motivated by the visual inspection of the spectrum, are partly subjective. Here, they serve as a mere illustration of the clustering approach prior to applying CA. Choosing the number of dimensions and clusters in data is a widely discussed problem in various clustering methods. In networked data, a common method is optimizing modularity^53^. A more in-depth discussion is beyond the scope of this paper, as the examples given in this paper are mainly for illustration purposes.

The insight that ECI is equal to the first axis of CA questions its interpretation as a measure of complexity (as pointed out also in Ref.^10^). Rather, analyses involving the ECI can be seen as a form of gradient analysis, revealing an underlying latent variable that is associated with GDPpc. The different interpretations presented in this paper may shed new light on the empirical results found within the literature on economic complexity, for example by providing an interpretation for the higher order axes and their eigenvalues. Furthermore, the observation that the complexity indices are a one-dimensional embedding of a similarity network unifies the complexity indices with the so-called ‘product space’, that is well known in the economic complexity literature^46^. According to the present interpretation of CA, the product complexity index (PCI) is simply a one-dimensional representation of the product space.

The literature on CA provides a rich set of tools^54^ that can be used in the context of economic complexity, including analysis of the contribution of countries or products to the position of each axis (‘which countries are the main contributors to ECI?’), measures for how well specific countries or products are represented by an axis (‘which countries are well represented by ECI’?), and ways to visualize additional points in the embedding space (‘how will a country move along the complexity rankings when adding some particular products?’).

## Discussion

In this paper, we provided an overview of different mathematical derivations that all lead to CA. We showed that CA is closely related to the spectral analysis of a similarity network inferred from a bipartite network defined by an incidence matrix. This leads to the observation that ordination, clustering and dimensionality reduction are just three sides of the same coin. Reviewing and bringing together the different mathematical derivations and their interpretations may guide practitioners in the application of CA to different datasets.

When applying CA, the eigenvalues corresponding to each axis are indicative of the correlation between row and column scores, as well as the variation explained by each CA axis. Axes corresponding to large eigenvalues may represent both a gradient underlying the data, and hold information on clustering structure in the similarity network. The distribution of the eigenvector components within an axis are suggestive of the appropriate interpretation: a continuous distribution suggests that an axis reflects a gradient underlying the data, whereas eigenvectors with a limited number of approximately constant values suggest that an axis holds information about the clusters in the similarity network.

The full spectrum of eigenvalues further provides information about the structure of the data. A spectrum containing multiple eigenvalues close to one, followed by a clear drop in the eigenvalues, may be indicative of a similarity network that contains multiple (weakly connected) clusters. In such a case, we propose to cluster the data prior to performing gradient analysis, and analyze potential gradients within each cluster separately. In the ecological example, this was done using the eigengap heuristic. In the economic complexity example, we manually determined the number of clusters based on the spectrum of eigenvalues, and showed that removing clusters that are weakly connected in the similarity network, along with some other higher order axes, may lead to clearer gradients, effectively removing outliers.

However, a formal way of distinguishing axes that represent a continuous gradient from axes that describe clustering structure is still lacking. This problem is very closely related to the question of how to determine the number of clusters in the spectral clustering approach. Although the use of the eigengap heuristic is common practice, it is based on intuition, and formalizing approaches like the eigengap heuristic is an ongoing topic of research^55^. Another possible way forward is to take into account the distribution of entries of the axes, in addition to the eigenvalues, to determine whether an axis represents a continuous gradient or whether instead approximately discrete values indicate clustering structure^56^.

Furthermore, we note that the usage of k-Means as a way to cluster the spectral representation of a network might be problematic, as it determines cluster labels by assuming spherical cluster shapes. As there is no underlying basis for assuming any cluster shape, given the abstract networks derived from e.g. ecological or economic data, further research is needed to understand the performance of other clustering algorithms in the context of CA. In particular, density-based clustering techniques such as DBSCAN^57^, which emphasize the similarity between nodes instead of partitioning a network, might be a promising step forward. In addition, exploring other dimensionality reduction techniques to obtain simplified representations of a data set, different from the spectral embedding discussed here, might be a promising way forward in the applications discussed in this paper.

Finally, we must notice also that, within the framework of CA, there is no clear-cut distinction between clustering and gradient analysis. As we have shown, both clustering and ordination can be seen as a way of identifying latent variables underlying the data. Especially in noisy data, a CA axis may be somewhere at an intermediate point between identifying clusters and representing a gradient. A principled way of distinguishing the different functions of CA axes would require an underlying theory, or a null-model, specific to the research question at hand, against which the results can be compared in order to select relevant axes. Lacking such models, the distinction between the two remains partly a matter of heuristics.

## Supplementary Information

Supplementary Information.
