# Supplementary material for: Correspondence analysis, spectral clustering and graph embedding: applications to ecology and economic complexity
Source: Sci Rep. 2021 Apr 26;11:8926. doi: 10.1038/s41598-021-87971-9 (PMC8076183; doi:10.1038/s41598-021-87971-9)
Supplement: Supplementary file 1 — Supplementary Information. [file 41598_2021_87971_MOESM1_ESM.pdf]

Supplementary Material of: Correspondence analysis, spectral clustering and graph embedding: applications to ecology and economic complexity.

Alje van Dam[1,2,+], Mark Dekker[2,3], Ignacio Morales-Castilla[4], Miguel Á. Rodríguez[4], David Wichmann[2,5], and Mara Baudena[6,1,2,4,*]

[+]Corresponding author: aljevdam@gmail.com
[*]Corresponding author: m.baudena@isac.cnr.it
[1]Copernicus Institute of Sustainable Development, Utrecht University, the Netherlands
[2]Centre for Complex Systems Studies, Utrecht University, the Netherlands
[3]Department of Information and Computing Sciences, Utrecht University, The Netherlands
[4]GloCEE - Global Change Ecology and Evolution Group - Department of Life Sciences, University of Alcalá, Spain
[5]Institute for Marine and Atmospheric Research Utrecht, Utrecht University, the Netherlands
[6]National Research Council of Italy - Institute of Atmospheric Sciences and Climate (CNR-ISAC), Turin, Italy

A Relation between normalized cut and correlation analysis

We show that the eigenvalues and eigenvectors of Eq. (2) that result from the correlation analysis are directly related to those that follow from minimizing the normalized cut (Eq. (3)). Recall that minimizing the normalized cut results in the generalized eigenproblem

$$(D_r - S_r)\mathbf{v} = \tilde{\lambda} D_r \mathbf{v}.$$

Pre-multiplying by D_r^{-1}, this can be rewritten as

$$\begin{aligned}(I - D_r^{-1}S_r)\mathbf{v} &= \tilde{\lambda}\mathbf{v}\\ D_r^{-1}S_r\mathbf{v} &= (1-\tilde{\lambda})\mathbf{v}\\ D_r^{-1}AD_c^{-1}A^T\mathbf{v} &= \lambda\mathbf{v},\end{aligned}$$

which shows that solutions to Eq. (2) are solutions to Eq. (3) with $\lambda = 1 - \tilde{\lambda}$.

B Constructing the country-product matrix

The data contains for every country how much of each product is has exported in the year 2016. To obtain a binary 'presence-absence' matrix, we consider whether a country exports a product with 'revealed comparative advantage' (RCA). The RCA index compares the share of a product within a countries' export portfolio to the global share of that product in world trade to evaluate whether a country exports more than expected by the global share. If q_{ij} denotes the exports of product j in country i (given in dollars), the RCA is defined as $RCA(i,j) = \frac{q_{ij}/\sum_j q_{ij}}{\sum_i q_{ij}/\sum_{i,j} q_{ij}}$. The matrix A is then defined as

$$A_{ij} = \begin{cases} 1 & \text{if } RCA(i,j) > 1 \\ 0 & \text{if } RCA(i,j) \leq 1, \end{cases}$$

C Country clusters

cluster	countries
2	Algeria, Angola, Brunei Darussalam, Chad, Congo, Congo (Democratic Republic of the), Equatorial Guinea, Gabon, Iraq, Kuwait, Libya, Nigeria, Papua New Guinea, Qatar, South Sudan, Turkmenistan, Venezuela
3	Afghanistan, Albania, American Samoa, Andorra, Anguilla, Antarctica, Antigua and Barbuda, Argentina, Armenia, Aruba, Australia, Austria, Azerbaijan, Bahrain, Bangladesh, Barbados, Belarus, Belgium, Belize, Benin, Bhutan, Bolivia, Bonaire, Bosnia and Herzegovina, Botswana, Bouvet Island, Brazil, British Indian Ocean Territory, Bulgaria, Burkina Faso, Burundi, Cabo Verde, Cambodia, Cameroon, Canada, Central African Republic, Chile, China, Christmas Island, Cocos (Keeling) Islands, Colombia, Comoros, Costa Rica, Croatia, Cuba, Curaçao, Cyprus, Czech Republic, Côte d'Ivoire, Denmark, Djibouti, Dominica, Dominican Republic, Ecuador, Egypt, El Salvador, Eritrea, Estonia, Eswatini, Ethiopia, Fiji, Finland, France, Gambia, Georgia, Germany, Ghana, Greece, Grenada, Guam, Guatemala, Guinea, Guinea-Bissau, Guyana, Haiti, Heard and McDonald Islands, Honduras, Hong Kong, Hungary, India, Indonesia, Iran, Ireland, Israel, Italy, Jamaica, Japan, Jordan, Kazakhstan, Kenya, Kyrgyzstan, Laos, Latvia, Lebanon, Lesotho, Liberia, Lithuania, Luxembourg, Macao, Madagascar, Malawi, Malaysia, Mali, Malta, Mauritius, Mexico, Moldova, Mongolia, Montenegro, Montserrat, Morocco, Mozambique, Myanmar, Namibia, Nauru, Nepal, Netherlands, New Zealand, Nicaragua, Niger, Niue, Norfolk Island, North Korea, North Macedonia, Northern Mariana Islands, Norway, Oman, Pakistan, Palestine, Panama, Paraguay, Peru, Philippines, Pitcairn, Poland, Portugal, Romania, Russian Federation, Rwanda, Saint Barthélemy, Saint Helena, Ascension and Tristan da Cunha, Saint Kitts and Nevis, Saint Lucia, Saint Pierre and Miquelon, Samoa, San Marino, Sao Tome and Principe, Saudi Arabia, Senegal, Serbia, Sierra Leone, Singapore, Slovakia, Slovenia, Somalia, South Africa, South Georgia and South Sandwich Islds., South Korea, Spain, Sri Lanka, St-Martin / St Maarten, Sudan, Suriname, Sweden, Switzerland, Syrian Arab Republic, Taiwan, Tajikistan, Tanzania, Thailand, Timor-Leste, Togo, Tokelau, Tonga, Trinidad and Tobago, Tunisia, Turkey, Turks and Caicos Islands, Uganda, Ukraine, United Arab Emirates, United Kingdom, United States Minor Outlying Islands, United States of America, Uruguay, Uzbekistan, Vatican City, Vietnam, Wallis and Futuna, Western Sahara, Yemen, Zambia, Zimbabwe
4	Bahamas, Bermuda, Cayman Islands, Cook Islands, Falkland Islands, Faroe Islands, French Polynesia, French Southern and Antarctic Lands, Gibraltar, Greenland, Iceland, Kiribati, Maldives, Marshall Islands, Mauritania, Micronesia, New Caledonia, Palau, Saint Vincent and the Grenadines, Seychelles, Solomon Islands, Tuvalu, Vanuatu, Virgin Islands (British)
